# An Effective Quality Control of Pharmacologically Active Volatiles of *Houttuynia cordata* Thunb by Fast Gas Chromatography-Surface Acoustic Wave Sensor

**DOI:** 10.3390/molecules200610298

**Published:** 2015-06-03

**Authors:** Se Yeon Oh

**Affiliations:** 1 Herbal Medicine Analytical Laboratory, KOSMO NF Co., Ltd., 28-8 Seongan-ro 13Gil, Gangdong-Gu, Seoul 134-849, Korea; E-Mail: syohkosmo62@hanmail.net; Tel.:+82-2477-3291; Fax: +82-2477-3281; 2 College of Science, Sejong University, 209 Neungdong-ro, Gwangjin-Gu, Seoul 143-747, Korea

**Keywords:** gas chromatography/surface acoustic wave sensor, *Houttuynia cordata* Thunb, fragrance pattern recognition, quality control

## Abstract

Fast gas chromatography-surface acoustic wave sensor (GC/SAW) has been applied for the detection of the pharmacological volatiles emanated from *Houttuynia cordata* Thunb which is from South Korea. *H. cordata* Thunb with unpleasant and fishy odors shows a variety of pharmacological activities such as anti-microbial, anti-inflammatory, anti-cancer, and insect repellent. The aim of this study is to show a novel quality control by GC/SAW methodology for the discrimination of the three different parts of the plant such as leaves, aerial stems, and underground stems for *H. cordata* Thunb. Sixteen compounds were identified. β-Myrcene, *cis*-ocimene and decanal are the dominant volatiles for leaves (71.0%) and aerial stems (50.1%). While, monoterpenes (74.6%) are the dominant volatiles for underground stems. 2-Undecanone (1.3%) and lauraldehyde (3.5%) were found to be the characteristic components for leaves. Each part of the plant has its own characteristic fragrance pattern owing to its individual chemical compositions. Moreover, its individual characteristic fragrance patterns are conducive to discrimination of the three different parts of the plant. Consequently, fast GC/SAW can be a useful analytical method for quality control of the different parts of the plant with pharmacological volatiles as it provides second unit analysis, a simple and fragrant pattern recognition.

## 1. Introduction

Perennial *Houttuynia cordata* Thunb (Saururaceae) is an important medicinal plant in Asia. Because *H. cordata* Thunb with unpleasant and fishy odors shows a variety of pharmacological activities. The chemical components of *H. cordata* Thunb comprise of volatiles, flavonoids, alkaloids, fatty acids, sterols, and polyphenolic acids [1]. Especially, its volatile components have many clinical benefits including anti-microbial, anti-inflammatory, anti-cancer, anti-fungal, anti-bacterial, and insect repellent [2]. Thus, the analysis of volatile constituents of *H. cordata* Thunb is of great interest in clinical therapy and food industries.

Conventionally, the analysis of volatiles from the plant material is preceded by isolation procedures including solvent extraction [3], steam distillation [4], and simultaneous distillation extraction [5], which involve excessive manipulation of the sample, a very costly, time-consuming procedure, low extraction efficiency, losses of some volatile compounds, and degradation of volatiles. Especially, the analysis of volatile compounds demands rapid and simple procedures, because volatile components are sensitive and decompose when exposed to new chemical components in air or light.

Since 1989, headspace solid-phase microextraction (HS-SPME) as a headspace sampling technique has been widely used for the analysis of volatile and semi-volatile compounds in plant, food, biological, and environmental fields. Headspace sampling has many advantages, the most important of which is the elimination of much of the interferences arising from the sample matrix. The merits of the headspace SPME method include solvent-free, smaller sample volumes, and shorter analysis time [6,7,8]. Also, volatile aromas are usually composed of complex mixtures of many volatiles; human sensory evaluation by trained panelists is important in aroma analysis. However, it has many limitations which involve a very expensive, time consuming procedure, and subjectiveness of experts. Finally, there is enormous demand for the development of an analytical method which provides rapid, simple, on-line measurements, non-destructive, low-cost procedures and rapid sensory information.

Consequently, one of such methods seems to be the electronic nose (eNose) which is rapid and has high sensitivity for the analysis of volatile components [9,10]. A piezoelectric 10 MHz multichannel quartz crystal microbalance (MQCM) based eNose behaves as an artificial receptor which can be compared to human olfactory systems. Furthermore, a remarkable sensitivity and selectivity is achieved while comparing fresh and dried herbs, even at the isomeric level e.g., α-pinene and β-pinene. Then, eNose method can be useful to determine the freshness, usability and shelf-life of herbs [11]. However, aroma analysis with the eNose has not always been successful. It is very sensitive to drift and lacks the possibility for identification of the different aroma components causing the signal change [12].

Since 1998, a new technique, fast gas chromatography with surface acoustic wave sensor (GC/SAW, zNose), called electronic nose based on the headspace sampling method provides second unit analysis, simple, low-cost procedure, real time detection, and rapid sensory information [13,14,15,16,17,18,19,20,21,22,23,24,25].

For the first time, a chemical sensor for organic vapor detection by coating a sensitive film on the surface of a SAW device was introduced by Wohltjen, H. and Dessy, R. in 1979 [26]. A various SAW sensors use mainly sensitive coatings film on the surface of a SAW sensor. The sensitive film can strongly adsorb a certain kind of vapors and almost does not adsorb other vapors. The importance of this kind of sensor is to coat a sensitive film with high selectivity and high adsorption capacity. Thus, they are applicable to measure the content of some special vapors [27].

In the fields of the various applications, including environmental pollutant monitoring, discrimination of botanical and geographical origin for organisms, detection of off-flavors in raw materials of food, diagnostic of a disease through breath emitted from patients, tracking out drugs and explosives, and the measurements of the change of aroma quality according to real-time for living-organisms, there is an enormous demand for sensors which have a wide working range to detect volatile organic compounds.

Finally, the SAW detector in zNose is a small miniature vapor chemical sensor used to detect volatile organic compounds in a wide range. The base material of a SAW device is an uncoated piezo-electric quartz crystal. This crystal is in contact with a thermoelectric element, which controls the temperature for cooling during vapor adsorption and for heating during desorption. The crystal operates by maintaining highly focused and resonant surface acoustic waves of 500 MHz on its surface. When an analyte adsorbs on the surface of the sensor, the frequency of the surface acoustic wave is altered, which in turn affects the detection signal and allows identification of the compounds.

Especially, the characteristic of simplicity of fast GC/SAW method does not demand extraction procedures and provides on-line measurements. The headspace vapor of sample is swept into the inlet via a pump, and then the vapor passes through the valve where the compounds are adsorbed onto the trap inside the system. Switching the valve to the injection process causes helium gas to flow backwards through the trap and onto the column [22,25]. Therefore, GC/SAW has diminished decomposition of plant materials, and minimized activity of enzyme. Especially, fast GC/SAW permits a high reproducibility and good sensitivity at the high picogram to nanogram level, making it possible to detect sensitive living volatile materials quantitatively [22]. Also, GC/SAW allows pattern recognition by a visual fragrance pattern, called a VaporPrint or chromatographic fingerprint derived from the frequency of a SAW sensor [20,21,22,23,24,25]. Chromatographic fingerprint technique has been internationally accepted as a useful means for quality control [28,29].

Therefore, fast GC/SAW method allows to monitor rapid change in volatile emission, and this method can be used in the field facilitating reliable ecological studies on living organism under undisturbed conditions in real time. The advantages and the method validation of GC/SAW, adaptability to a variety of applications were reported in my previous publications [20,21,22,23,24,25].

Some papers were published about the analysis of chemical compositions of essential oil for *H. cordata* Thunb which are originated from China by GC-MS [29,30]. Also, the paper reported on the analysis of volatiles of *H. cordata* Thunb which are from China by HS-SPME-GC-MS [31]. However, no paper has reported on the analysis of volatile compounds of *H. cordata* Thunb which are from South Korea by using fast GC/SAW and HS-SPME-GC-MS.

The aim of this study is to show a novel quality control by fast GC/SAW methodology for the discrimination of the three different parts of the plant such as leaves, aerial stems, and underground stems for *H. cordata* Thunb which are from South Korea. In addition, volatile composition profile data for *H. cordata* Thunb by GC/SAW were analyzed by PCA. Also, HS-SPME-GC-MS method was employed to confirm the identification of volatile compounds of *H. cordata* Thunb and compared to GC/SAW.

## 2. Results and Discussion

### 2.1. Identification of Pharmacologically Active Volatiles of Houttuynia cordata Thunb by GC/SAW

**Figure 1:**
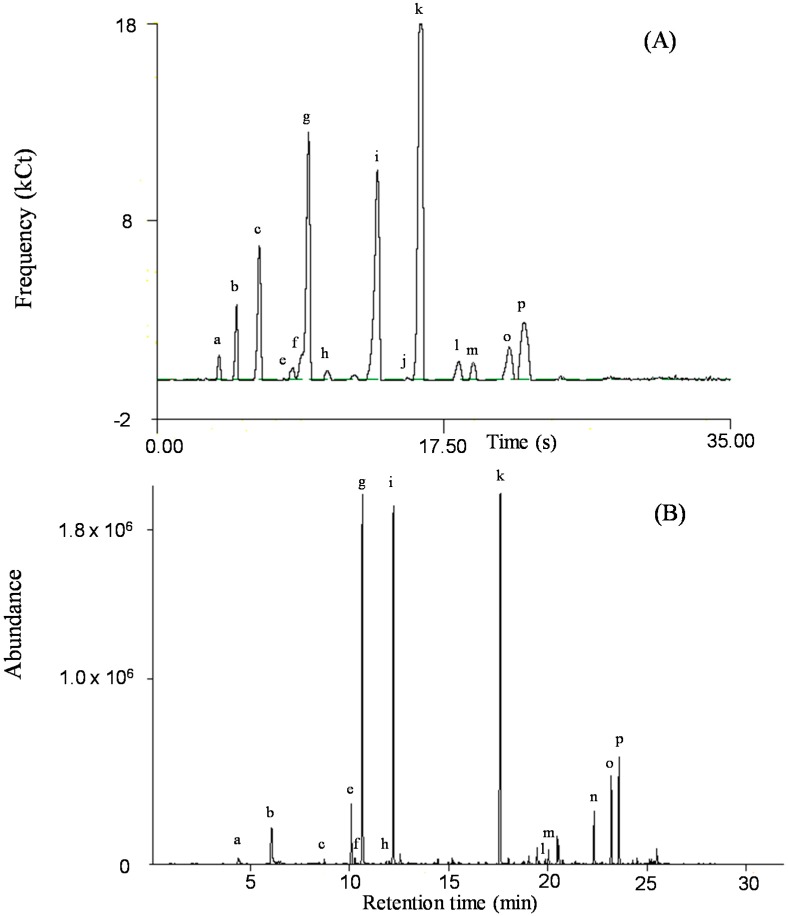
Comparison of chromatograms of leaves for *Houttuynia cordata* Thunb (Anmyeon Island, South Korea) by fast GC/SAW (**A**) and HS-SPME-GC-MS (**B**). Peak identities: a, *cis*-3-Hexenal; b, 3-Hexen-1-ol; c, α-Pinene; d, Camphene; e, Sabinene; f, β-Pinene; g, β-Myrcene; h, Limonene; i, *cis*-Ocimene; j, α-Terpinolene; k, Decanal; l, Bornyl acetate; m, 2-Undecanone; n, Geranyl acetate; o, Lauraldehyde; p, β-Caryophyllene.

The profiles of pharmacologically active volatiles of *H. cordata* Thunb were obtained using fast GC/SAW. The frequency of the SAW sensor is altered when an analyte adsorbs on the surface of the sensor, which affects the detection signal in direct proportion to the amount of condensate. Figure 1A shows chromatograms of the volatile compounds of leaves for *H. cordata* Thunb which are from Anmyeon Island, South Korea. The area of each peak is expressed in frequency counts (Cts).

Each volatile compound detected by GC/SAW was identified by a comparison with authentic standards and GC-MS analysis. Their relative proportions (% total amounts) of leaves, aerial stems, and underground stems for *H. cordata* Thunb which are from Anmyeon Island, South Korea are summarized in Table 1.

**Table 1:** Composition and identification of volatiles of leaves, aerial stems, and underground stems for *Houttuynia cordata* Thunb by fast GC/SAW and HS-SPME-GC-MS.

Peak No.	Compound	*Houttuynia cordata* Thunb							
		Leaves			Aerial Stems			Underground Stems	
		GC/SAW	HS-SPME	GC/SAW		HS-SPME	GC/SAW		HS-SPME
a	*cis*-3-Hexenal	1.1(14.81)	0.3 (3.85)	2.1(12.78)		-	0.9(3.71)		-
1	Hexanal	-	0.3(31.33)	-		-	-		-
2	2-Hexenal	-	1.2(22.09)	-		-	-		-
b	3-Hexen-1-ol	3.5(9.75)	2.5(10.80)	0.2(4.68)		1.1(6.67)	-		-
3	α-Thujene	-	0.1(8.93)	-		0.2(9.01)	-		0.2(5.56)
c	α-Pinene	8.3(3.28)	0.2(6.48)	4.2(4.39)		5.3(4.31)	-		15.3(6.10)
d	Camphene	-	-	0.6(3.14)		1.8(3.16)	1.9(17.06)		1.3(3.99)
e	Sabinene	0.8(13.09)	3.0(4.64)	2.1(9.92)		4.5(4.92)	3.2(1.49)		6.9(2.23)
f	β-Pinene	0.3(14.28)	0.3(5.86)	-		4.0(7.11)	1.5(2.81)		28.8(3.07)
g	**β-Myrcene**	15.8(5.71)	26.2(8.39)	23.3(9.32)		48.1(10.76)	33.9(10.50)		14.3(7.21)
4	δ-3-Carene	-	0.1(30.34)	-		-	-		-
5	α-Terpinene	-	0.1(12.78)	-		0.1(8.98)	-		0.1(7.95)
6	*p-*Cymene	-	0.1(9.81)	-		0.4(6.23)	-		0.2(4.61)
h	**Limonene**	0.6(12.57)	0.1(8.61)	2.3(7.47)		4.0(5.13)	17.4(8.06)		9.4(4.33)
i	***cis*-Ocimene**	18.4(5.93)	18.2(2.33)	11.1(14.55)		6.6(4.63)	15.9(16.93)		0.2(2.15)
7	*trans*-Ocimene	-	0.5(21.92)	-		0.2(15.73)	-		0.2(13.76)
8	γ-Terpinene	-	0.1(2.81)	-		0.2( 3.45)	-		0.2(1.09)
9	*trans*-Sabinene hydrate	-	-	-		-	-		t
j	α-Terpinolene	0.1(4.02)	-	0.9(15.06)		-	0.8(14.96)		0.5(5.67)
10	*cis*-Sabinene hydrate	-	-	-		-	-		t
11	Nonanal	-	0.2(11.95)	-		0.1(13.32)	-		t
12	2,6-Dimethyl-1,3,5,7-octatetraene	-	0.1(5.35)	-		-	-		-
13	*neo-allo*-Ocimene	-	0.3(5.30)	-		0.1(3.94)	-		-
14	1-Nonanol	-	0.1(9.66)	-		0.1(5.31)	-		0.1(6.11)
15	Terpinene-4-ol	-	0.1(10.14)	-		0.1(8.83)	-		0.1(4.31)
k	**Decanal**	36.8(11.58)	26.8(49.82)	15.7(15.00)		1.4(30.23)	1.2(1.46)		0.1(15.36)
16	α-Fencyl acetate	-	-	-		-	-		t
17	β-Cyclocitral	-	0.3(24.19)	-		0.1(19.12)	-		0.3(17.42)
18	Thymyl methyl ether	-	-	-		-	-		t
19	Citral	-	-	-		-	-		0.1(9.53)
20	1-Decanol	-	0.8(50.12)	-		0.2(35.29)	-		-
l	**Bornyl acetate**	1.7(6.75)	0.2(12.92)	17.4(8.06)		9.3(9.54)	14.6(9.16)		7.2(6.63)
m	**2-Undecanone**	1.3(10.49)	0.7(9.56)	-		0.7(8.90)	-		2.3(7.19)
21	Undecanal	-	1.3(1.66)	-		0.3(4.73)	-		0.1(3.71)
22	α-Cyclogeranyl acetate	-	0.9(26.34)	-		0.4(17.71)	-		0.8(15.03)
23	5-Methyl-3-(1-methyl-ethenyl)cyclohexene	-	0.2(20.98)	-		0.1(19.01)	-		0.3(13.77)
24	1-Methyl-3-(1-methyl-ethenyl)cyclohexene	-	0.1(16.37)	-		0.1(25.93)	-		0.2(15.79)
25	α-Terpinenyl acetate	-	-	-		0.1(23.01)	-		0.4(19.04)
26	Neryl acetate	-	t	-		0.1(20.01)	-		0.2(15.90)
n	Geranyl acetate	-	2.5(34.74)	10.4(2.45)		1.6(23.63)	3.1(7.94)		1.1(29.07)
27	β-Elemene	-	0.1(27.35)	-		0.1(26.17)	-		0.1(21.45)
o	**Lauraldehyde**	3.5(7.69)	4.4(50.59)	-		0.4(35.12)	-		0.1(43.17)
p	β-Caryophyllene	7.8(8.73)	5.5(34.50)	9.7(9.31)		6.6(40.31)	5.6(11.87)		5.1(30.01)
28	Aromadendrene	-	t	-		-	-		-
29	β-Farnesene	-	0.2(48.56)	-		0.2(37.81)	-		0.1(40.48)
30	α-Humulene	-	0.3(37.04)	-		0.3(40.89)	-		0.3(33.67)
31	Azulene	-	-	-		0.1(26.38)	-		0.4(22.93)
32	Germacrene-D	-	0.2(55.55)	-		0.1(43.10)	-		t
33	α-Farnesene	-	0.2(54.50)	-		-	-		-
34	Aromadendrene	-	0.1(41.41)	-		0.1(47.17)	-		2.2(38.38)
35	*allo*-Aromadendrene	-	0.2(46.97)	-		0.1(39.05)	-		-
36	Bicyclogermacrene	-	0.9(56.26)	-		-	-		-
37	α-Selinene	-	-	-		0.7(41.42)	-		0.7(42.59)
38	δ-Cadinene	-	t	-		-	-		-
39	Caryophyllene oxide	-	-	-		-	-		0.1(27.67)

(-) Not detected; t, <0.05%. % Figures are their relative proportions as percent of total area, %(RSD), *n =* 3. The characteristic compounds are indicated in bold. *H. cordata* Thunb which are from Anmyeon Island, South Korea was used. Column: DB-624 (6% cyanopropyl phenyl polydimethylsiloxane, 1 m × 0.25 mm × 0.25 µm) fused silica capillary column was used by GC/SAW. Ultra 2 (5% phenyl polydimethylsiloxane, 25 m × 0.25 mm × 0.33 µm) fused silica capillary column was used by HS-SPME-GC-MS. Sampling time: The headspace vapor of sample was swept into the trap inside the system at 25 °C for 1 s by GC/SAW. The SPME fiber was exposed to the headspace above the sample vial at 25 °C for 1 h by HS-SPME-GC-MS.

Sixteen compounds were identified. β-Myrcene, *cis*-ocimene and decanal, which comprise 71.0% of the total amounts, are the dominant volatile components of leaves of *H. cordata* Thunb. 2-Undecanone and lauraldehyde were found to be the characteristic components comprising 1.3% and 3.5% of the total amounts, respectively. Monoterpenes such as α–pinene, sabinene, β-pinene, and α-terpinolene as minor constituents were also found. In addition, the amount of limonene and bornyl acetate varied from low to moderate concentrations for leaves, aerial stems, and underground stems.

It was reported that the principal component in the essential oil of this medicinal plant, decanoyl acetaldehyde, is known to have pharmacological effects, but it is unstable and easily converted into 2-undecanone by oxidation and decarboxylation [2,31]. 2-Undecanone also known as methyl nonyl ketone is contributed as an insect repellent for strong odor. Also, unpleasant and fishy odors of *H. cordata* Thunb is caused by decanoyl acetaldehyde and lauraldehyde [32].

Monoterpenes (β-myrcene, *cis*-ocimene, and limonene), decanal, geranyl acetate, and bornyl acetate have pharmacological properties such as antimicrobial, anti-fungal, anti-bacterial. In addition, limonene has an anti-cancer effect. Lauraldehyde and 2-undecanone have anti-virus properties [2,33].

In aerial stems for *H. cordata* Thunb, β-myrcene, *cis*-ocimene and decanal, which comprise 50.1% of the total amounts, are also the dominant volatile components. 2-Undecanone and lauraldehyde were not found. The concentration of bornyl acetatate (17.4%) is considerably higher than that of leaves (1.7%) and geranyl acetate (10.4%) was found, as indicated in Table 1. The total amounts of pharmacologically active volatiles of aerial stems for *H. cordata* Thunb are four times lower than those of leaves or underground stems.

In underground stems for *H. cordata* Thunb, monoterpenes involving β-myrcene, *cis*-ocimene, camphene, sabinene, β-pinene, limonene, and α-terpinolene which comprise 74.6% of the total amounts, are also the dominant volatile components. While the level of decanal (1.2%) was substantially lower than that of leaves (36.8%) or aerial stems (15.7%). Interestingly, the quantity of limonene (17.4%) is remarkably increased than that of leaves (0.6%) or aerial stems (2.3%), the concentration of bornyl acetatate (14.6%) is considerably higher than that of leaves (1.7%), and geranyl acetate (3.1%) was found. 2-Undecanone and lauraldehyde were not found like aerial stems, as shown in Table 1.

It is interesting to note that the components found in leaves, aerial stems, and underground stems of *H. cordata* Thunb are almost the same. The only difference is the proportion of such components present. Such differences lead to the discrimination of three different parts of the plant such as leaves, aerial stems, and underground stems of *H. cordata* Thunb.

It was reported that 2-undecanone (22.2%) and decanoyl acetaldehyde (7.2%) are predominant components for *H. cordata* Thunb which are from Jiangxi Province, China by using HS-SPME-GC-MS [31]. The populations of *H. cordata* Thunb which are from Sichuan and Chongqing Province, China have been divided into two chemotypes. Chemotype myrcene (M) comprise mainly monoterpenes and inversely chemotype decanal (D) has a lower content of monoterpenes [30].

### 2.2. Comparison of GC/SAW and HS-SPME-GC-MS method for H. cordata Thunb

The compositions of the pharmacologically active volatile compounds of *H. cordata* Thunb which are from Anmyeon Island, South Korea extracted by HS-SPME using CAR/DVB/PDMS fiber and then analyzed by GC-MS are presented in Table 1. Its GC-MS total ion chromatogram is shown in Figure 1B. The alphabetic numbers of the peaks shown in Figure 1B correspond to the numbers indicated in the GC/SAW chromatograms (Figure 1A). Fifty-five compounds were detected by HS-SPME-GC-MS. Major compounds were identified by a comparison of the retention times and mass spectra of authentic standards. Minor compounds were tentatively identified by matching with their mass spectra of Wiley/NIST Library in GC-MS software.

HS-SPME-GC-MS have been used widely for the analysis of volatiles as high resolution capacity and wide linear dynamic range. Also it was reported that the calculated limit of detection (LOD) varied from 0.1–100 ng [34]. In my previous paper, Multi-point calibration was performed for the three representative characteristic components of *S. vulgaris variginata* by GC/SAW. The calibration curves of the analytes showed good linearity over the range of 5.72~175 ng for benzyl methyl ether, 5.04~300 ng for phenylacetaldehyde, and 49.4~1235 ng for α-pinene. The obtained LODs, calculated at S/N = 3, varied from 500 pg (phenylacetaldehyde) to 13 ng (α-pinene) (Table 2) [22].

In this study, the HS-SPME-GC-MS can detect 55 compounds in the *H. cordata* Thunb which are from Anmyeon Island, South Korea, far more than what the GC-SAW could detect (16 compounds). The difference of trapping time used in these two methods in order to set the proper conditions leads to these results. The headspace vapor of sample was swept into the trap inside the system at 25 °C for 1 s by GC/SAW, whereas the SPME fiber was exposed to the headspace above the sample vial at 25 °C for 1 h by HS-SPME-GC-MS.

As shown in Table 1, β-myrcene, *cis*-ocimene and decanal, which comprise 71.2% of the total amounts, are the dominant volatile components of leaves of *H. cordata* Thunb, just as GC/SAW (71.0%). 2-Undecanone and lauraldehyde, as the characteristic components in *H. cordata* Thunb, also comprised 0.7% and 4.4% of the total amounts, respectively.

In aerial stems for *H. cordata* Thunb, β-myrcene, *cis*-ocimene and decanal, which comprise 56.1% of the total amounts, are also the dominant volatile components. 2-Undecanone (0.7%) and lauraldehyde (0.4%) were found at low concentrations. The concentration of bornyl acetatate (9.3%) is considerably higher than that of leaves (0.2%) and geranyl acetate (1.6%) was found, as GC/SAW. The total amounts of pharmacologically active volatiles of aerial stems for *H. cordata* Thunb are lower 1.5 times than those of leaves.

In underground stems for *H. cordata* Thunb, monoterpenes involving β-myrcene, *cis*-ocimene, α-thujene, α-pinene, camphene, sabinene, β-pinene, α-terpinene, limonene, *trans*-ocimene, γ-terpinene, α-terpinolene,5-methyl-3-(1-methyl-ethenyl)cyclohexene,1-methyl-3-(1-methylethenyl)cyclohexene which comprise 77.9% of the total amounts, are also the dominant volatile components. While the level of decanal (0.1%) was substantially lower than that of leaves (26.8%), such as GC/SAW. Also, the quantity of limonene (9.4%) is remarkably increased than that of leaves (0.1%), the concentration of bornyl acetatate (7.2%) is considerably higher than that of leaves (0.2%) and geranyl acetate (1.1%) was found, as GC/SAW. 2-Undecanone (2.3%) and lauraldehyde (0.1%) were found a little, as shown in Table 1.

As these results show, the dominant components and analytical tendency for *H. cordata* Thunb detected by GC/SAW and HS-SPME-GC-MS are similar, but there is a difference of abundance ratios owing to the difference of extraction efficiency and response factor between these two methods.

In my previous paper, it was reported that the ability for classification among species of completely different chemotypes by HS-SPME-GC-MS is good enough, but the classification of same chemotypes species which are from different geographical origin in same country, original species and its variety, an air-drying term for 13 days and 16 months appear much lower than GC/SAW [23].

**Table 2:** Composition and identification of volatiles of leaves, aerial stems, and underground stems for five different geographical origins of *Houttuynia cordata* Thunb by fast GC/SAW.

Peak No.	Compound	*Houttuynia cordata* Thunb														
		Leaves					Aerial Stems					Underground Stems				
		A	B	C	D	E	A′	B′	C′	D′	E′	A′′	B′′	C′′	D′′	E′′
a	*cis*-3-Hexenal	1.1(10.60)	1.1(14.81)	0.8(12.87)	0.9(8.49)	1.0(3.83)	2.1(6.97)	2.1(12.78)	1.7(13.66)	2.4(8.47)	1.5(16.69)	0.7(9.15)	0.9(3.71)	0.4(16.13)	0.6(3.47)	0.5(10.11)
b	3-Hexen-1-ol	4.9(7.78)	3.5(9.75)	2.9(17.89)	3.4(2.03)	4.0(4.09)	0.2(4.55)	0.2(4.68)	0.2(3.76)	0.3(11.26)	0.3(18.23)	-	-	-	-	-
c	α-Pinene	6.1(0.81)	8.3(3.28)	8.3(7.52)	11.7(1.55)	8.2(1.23)	2.2(1.71)	4.2(4.39)	3.0(7.47)	3.2(13.94)	2.7(2.36)	-	-	-	-	-
d	Camphene	-	-	-	-	-	0.9(4.05)	0.6(3.14)	0.5(9.35)	0.6(12.61)	0.5(10.25)	1.8(0.29)	1.9(17.06)	2.3(3.52)	2.3(13.74)	1.7(6.24)
e	Sabinene	0.6(11.11)	0.8(13.09)	0.7(14.73)	1.3(9.21)	0.7(5.68)	2.0(11.70)	2.1(9.92)	1.7(12.31)	1.6(8.98)	1.7(4.95)	3.1(4.29)	3.2(1.49)	2.8(2.67)	3.6(4.34)	3.2(5.07)
f	β-Pinene	-	0.3(14.28)	0.5(13.00)	0.5(4.92)	0.5(5.46)	0.8(13.19)	-	-	-	-	1.9(6.45)	1.5(2.81)	4.2(4.61)	3.6(8.53)	2.8(7.74)
g	**β-Myrcene**	21.3(3.58)	15.8(5.71)	13.5(10.59)	17.5(6.79)	18.9(0.80)	26.3(12.64)	23.3(9.32)	20.7(11.75)	26.7(0.81)	22.9(7.43)	38.0(13.26)	33.9(10.50)	46.6(7.85)	36.9(2.58)	36.3(2.65)
h	**Limonene**	0.3(14.29)	0.6(12.57)	0.5(3.27)	0.8(17.17)	0.6(13.65)	3.5(12.24)	2.3(7.47)	2.2(3.39)	2.7(0.81)	2.4(5.29)	18.6(10.09)	17.4(8.06)	17.3(2.87)	17.5(2.58)	16.4(3.32)
i	***cis*-Ocimene**	19.6(1.03)	18.4(5.93)	16.7(7.82)	22.6(5.42)	16.9(1.51)	14.6(15.36)	11.1(14.55)	14.2(11.92)	16.1(4.85)	14.3(10.68)	15.0(9.17)	15.9(16.93)	11.9(2.32)	15.0(6.70)	14.9(5.92)
j	α-Terpinolene	-	0.1(4.02)	0.2(14.28)	0.3(7.87)	0.2(13.62)	0.9(8.02)	0.9(15.06)	0.3(22.04)	0.3(13.04)	0.7(6.02)	0.3(9.90)	0.8(14.96)	0.5(8.52)	0.6(11.12)	0.4(18.87)
k	**Decanal**	31.9(4.56)	36.8(11.58)	41.9(8.65)	29.9(0.92)	36.8(6.43)	8.7(7.28)	15.7(15.00)	29.4(8.37)	21.4(5.83)	20.7(2.13)	0.4(10.27)	1.2(1.46)	1.0(10.34)	1.3(8.76)	2.6(8.29)
l	**Bornyl acetate**	3.1(5.87)	1.7(6.75)	1.1(9.49)	1.1(2.23)	1.8(2.87)	16.9(3.35)	17.4(8.06)	10.1(7.20)	9.9(3.98)	13.6(0.53)	12.4(8.67)	14.6(9.16)	8.3(6.38)	11.7(8.46)	12.8(5.82)
m	**2-Undecanone**	0.6(14.19)	1.3(10.49)	1.9(11.47)	1.2(4.74)	0.5(12.73)	-	-	-	-	-	-	-	-	-	-
n	Geranyl acetate	-	-	-	-	-	9.9(2.81)	10.4(2.45)	8.4(8.11)	7.9(10.80)	8.2(1.11)	2.2(6.82)	3.1(7.94)	1.9(3.43)	2.0(1.57)	2.6(2.17)
o	**Lauraldehyde**	3.3(2.98)	3.5(7.69)	3.8(1.54)	3.7(3.76)	3.2(3.78)	-	-	-	-	-	-	-	-	-	-
p	β-Caryophyllene	7.2(2.62)	7.8(8.73)	7.2(6.65)	5.1(2.47)	6.7(10.04)	11.0(0.92)	9.7(9.31)	7.6(5.32)	6.9(4.22)	10.5(5.07)	5.6(5.30)	5.6(11.87)	2.8(4.20)	4.9(9.60)	5.8(3.73)

(-) not detected. % Figures are their relative proportions as percent of total amount (Cts), %(RSD), *n* = 3. The characteristic compounds are indicated in bold. Column: DB-624 (6% cyanopropyl phenyl polydimethylsiloxane, 1 m × 0.25 mm × 0.25 µm) fused silica capillary column was used. Samples of wild *H. cordata* Thunb were collected from Ulreung island(A,A′,A′′), Anmyeon island(B,B′,B′′), Jeju island(C,C′,C′′), Hampyeong(D,D′,D′′), and Haenam(E,E′,E′′) in South Korea.

### 2.3. Discrimination and Quality Control of H. cordata Thunb by GC/SAW and Principal Component Analysis for GC/SAW Responses

**Figure 2:**
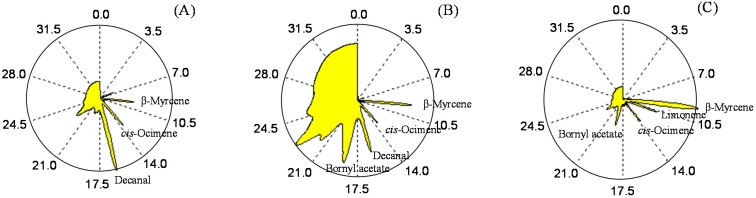
Comparison of fragrance patterns for leaves (**A**), aerial stems (**B**), and underground stems (**C**) of *Houttuynia cordata* Thunb (Anmyeon Island, South Korea) by fast GC/SAW.

GC/SAW allows pattern recognition by a visual fragrance pattern, called a VaporPrint or chromatographic fingerprint derived from the frequency of a SAW sensor [22,23,24,25]. Chromatographic fingerprint technique has been internationally accepted as a useful means for the quality control [28,29]. This image is created by transforming the time variable to a radial angle with the beginning and end of the analysis [22,23,24,25]. Figure 2A–C show the fragrance patterns of leaves, aerial stems, and underground stems for *H. cordata* Thunb which are from Anmyeon Island, South Korea. As shown in Figure 2A–C, each part of plant has own characteristic fragrance pattern owing to its individual chemical compositions. Moreover, its individual characteristic fragrance patterns are conducive to discrimination of the three different parts of plant such as leaves, aerial stems, and underground stems.

**Figure 3:**
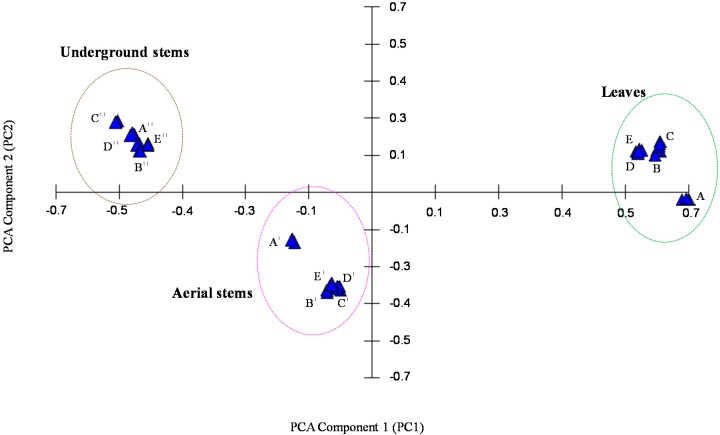
Principal component analysis for GC/SAW responses of leaves, aerial stems, and underground stems of *Houttuynia cordata* Thunb. (A) leaves, (A′) aerial stems, (A′′) underground stems, Ulreung Island, South Korea ; (B) leaves, (B′) aerial stems, (B′′) underground stems, Anmyeon Island, South Korea; (C) leaves, (C′) aerial stems, (C′′) underground stems, Jeju Island, South Korea; (D) leaves, (D′) aerial stems, (D′′) underground stems, Hampyeong, South Korea; (E) leaves, (E′) aerial stems, (E′′) underground stems, Haenam, South Korea.

Table 2 shows their relative proportions (% total amounts) for leaves, aerial stems, and underground stems of *H. cordata* Thunb which are from five different geographical origins in South Korea. To classify leaves, aerial stems, and underground stems, GC/SAW data profiles of 15 samples (Table 2) for *H. cordata* Thunb were analyzed by PCA. To display the points in two principal components, PC 1 and PC 2 (first and second principal components) were chosen to represent the information. Figure 3 shows a principal component projection plot of PC 1 and PC 2 of leaves, aerial stems, and underground stems for *H. cordata* Thunb (Ulreung Island, Anmyeon Island, Jeju Island, Hampyeong, and Haenam, South Korea). PC 1 and PC 2 reflected 96% of the total variance. As a result, a good classification among leaves, aerial stems, and underground stems for *H. cordata* Thunb was obtained. These results show that the three different parts of plant such as leaves, aerial stems, and underground stems can be discriminated easily using GC/SAW, as shown in Figure 3.

In my previous papers, it was reported that fast GC/SAW is an effective tool for species identifications and quality control [23,24,25].

## 3. Experimental Section

### 3.1. Materials

Samples of wild *H. cordata* Thunb were collected from Ulreung Island, Anmyeon Island, Jeju Island, Hampyeong, and Haenam in South Korea. The fresh plant materials including its leaves, aerial stems, and underground stems were prepared. All standard chemicals of analytical grade were purchased from Sigma-Aldrich (St. Louis, Mo, USA) and Tokyo Kasei (Nihonbashi, Tokyo Japan). Organic solvents of a chromatographic grade were obtained from J .T. Baker. The commercially available carboxen-divinylbenzene-polydimethylsiloxane (CAR-DVB-PDMS) SPME fiber (film thickness, 50/30 µm) was purchased from Supelco (Bellefonte, PA, USA) and used.

### 3.2. GC/SAW (zNose) Description

Fast GC/SAW (4100 vapor analyzer, Electronic Sensor Technology, New Bury Park, USA) composed with the fast gas chromatograph, a fast response integrating surface acoustic wave sensor detector, a pre-concentrating trap containing Tenax adsorbent, a short GC column is used to detect vapors of the volatile and semi-volatile organic compounds. The SAW sensor consists of an uncoated 500 MHz acoustic resonator, bonded to a thermoelectric heat pump to heat or cool the quartz substrate. The electrode material is gold. The GC/SAW is especially sensitive at the high pictogram to nanogram level [22].

### 3.3. GC/SAW Analytical Conditions and Procedure

About 1.0 g of each fresh wild *H. cordata* Thunb sample was weighed into a 40 mL glass vial sealed with a screw cap containing a Teflon/silicone septa. The capped vial was kept to equilibrate with the headspace vapor at 26 °C for 1 h just before analysis.

The headspace vapor of sample was swept into the trap inside the system for 1 s. The trap was heated rapidly to 200 °C to desorb and transfer the volatiles to the analytical column. GC column was heated from 32–120 °C at a rate of 3 °C/s. Helium (99.999%) was used as a carrier gas at 4.4 mL/min (0.07 mL/s). 6% Cyanopropyl phenyl polydimethylsiloxane (DB-624, J & W Scientific, Folsom, CA, USA, 1 m × 0.25 mm i.d., 0.25 µm film thickness) fused silica capillary column was used. The set-up temperatures were at 30 °C for sensor, 130 °C for inlet port, and 110 °C for valve. Triplicate measurements per sample were carried out. All analytical procedures were completed within 30 s. The shorter total time-to-result per sample allows several replicated analyses of a sample.

### 3.4. Headspace Solid-Phase Microextraction

About 2.0 g of fresh wild *H. cordata* Thunb sample was placed in a 30-mL vial sealed with an aluminum cap containing a Teflon/silicone septa. The capped vial was kept to equilibrate at 26 °C for 1 h before HS-SPME sampling. The carboxen-divinylbenzene-polydimethylsiloxane (CAR-DVB-PDMS) SPME fiber (film thickness, 50/30 µm) was used because it was most efficient among the various types of fiber for most volatile organic compounds [6,35]. The SPME fiber was exposed to the headspace above the *H. cordata* Thunb sample vial at 26 °C for 1 h. After adsorption, the SPME fiber was retracted from the sample vial and immediately inserted into the injection port of the GC-MS where thermal desorption was performed at 240 °C for 1 min.

### 3.5. GC-MS Analysis

All experiments were performed on a Hewlett-Packard 6890 Series GC system with an Agilent 5973 N Mass Selective Detector (Agilent Technologies, Wilmington, DE, USA) equipped with 5% phenyl polydimethylsiloxane (Ultra 2 column, Agilent, 30 m × 0.25 mm i.d., 0.25 µm film thickness). The oven temperature was initially maintained at 50 °C for 3 min and then programmed to 220 °C for 5 min at a rate 5 °C/min. Temperatures of the injector, transfer line, ion source, and quadrupole were set to 240 °C, 280 °C, 230 °C, and 150 °C, respectively. Helium (99.999%) was used as carrier gas at 1.0 mL/min under split mode (split ratio 1:30). The mass spectrometer was run in the electron impact (EI) mode with electron energy at 70 eV, scanning the 50.0–400.0 amu.

## 4. Conclusions

Fast GC/SAW provided sufficiently a novel quality control for the discrimination of the three different parts of plant such as leaves, aerial stems, and underground stems for *H. cordata* Thunb which are from South Korea. In addition, by PCA, leaves, aerial stems, and underground stems of *H. cordata* Thunb could be clearly discriminated from each other. Therefore, it is believed that GC/SAW can be a useful analytical method for quality control of the different parts of a plant with pharmacologically active volatiles as it provides second unit analysis, and a simple fragrance pattern recognition.
